# Enhancing Sealing Performance Predictions: A Comprehensive Study of XGBoost and Polynomial Regression Models with Advanced Optimization Techniques

**DOI:** 10.3390/ma18102392

**Published:** 2025-05-20

**Authors:** Weiru Zhou, Zonghong Xie

**Affiliations:** School of Aeronautics and Astronautics, Sun Yat-sen University, Shenzhen 518107, China; zhouwr5@mail2.sysu.edu.cn

**Keywords:** motor, sealing structure, polynomial regression, XGBOOST, simulated annealing algorithm, multi-objective particle swarm optimization algorithm

## Abstract

Motors, as the core carriers of pollution-free power, realize efficient electric energy conversion in clean energy systems such as electric vehicles and wind power generation, and are widely used in industrial automation, smart home appliances, and rail transit fields with their low-noise and zero-emission operating characteristics, significantly reducing the dependence on fossil energy. As the requirements of various application scenarios become increasingly complex, it becomes particularly important to accurately and quickly design the sealing structure of motors. However, traditional design methods show many limitations when facing such challenges. To solve this problem, this paper proposes hybrid models of machine learning that contain polynomial regression and optimization XGBOOST models to rapidly and accurately predict the sealing performance of motors. Then, the hybrid model is combined with the simulated annealing algorithm and multi-objective particle swarm optimization algorithm for optimization. The reliability of the results is verified by the mutual verification of the results of the simulated annealing algorithm and the particle swarm optimization algorithm. The prediction accuracy of the hybrid model for data outside the training set is within 2.881%. Regarding the prediction speed of this model, the computing time of ML is less than 1 s, while the computing time of FEA is approximately 9 h, with an efficiency improvement of 32,400 times. Through the cross-validation of single-objective optimization and multi-objective optimization algorithms, the optimal design scheme is a groove depth of 0.8–0.85 mm and a pre-tightening force of 80 N. The new method proposed in this paper solves the limitations in the design of motor sealing structures, and this method can be extended to other fields for application.

## 1. Introduction

**Figure 1:**
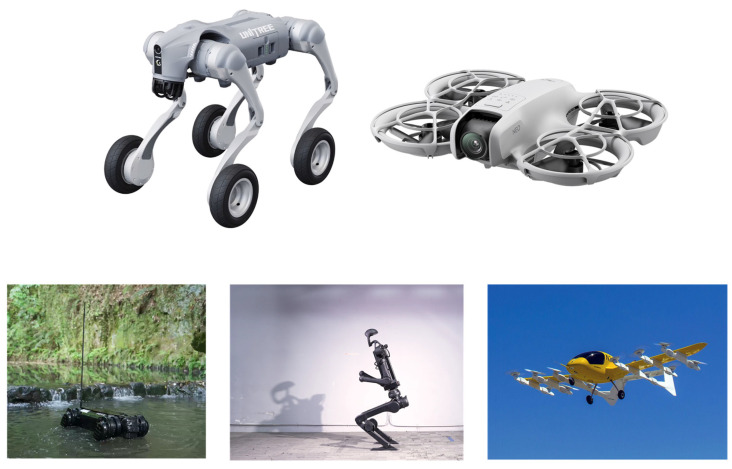
The application of motors.

Motors, with the high-efficiency conversion characteristics, can increase the efficiency of converting electrical energy into mechanical energy by over 90% [1]. Through intelligent control technologies such as variable frequency speed regulation and energy recovery, it can achieve precise speed and torque adjustment. The low-noise operation (<50 decibels) and zero-emission features significantly reduce noise and air pollution in industrial and living scenarios. Meanwhile, the brushless design and corrosion-resistant sealing structures (such as O-rings and stainless-steel housings) ensure stable and durable operation in harsh environments. In addition, motors have a compact structure and low maintenance costs (such as no need for carbon brushes and simplified mechanical components), and strong adaptability. Motors are widely used in fields such as electric vehicles, humanoid robots, drones, eVTOL, medical equipment, smart home appliances, and industrial automation as Figure 1 shows [2]. These advantages not only reduce dependence on fossil fuels but also contribute to reducing billions of tons of carbon emissions annually, promoting the global carbon neutrality process and sustainable development. Motors provide core support for humanity to achieve green energy transformation and intelligent production.

A good sealing performance is the foundation for the reliable operation of motors. By preventing the intrusion of external dust, water vapor, and chemicals, it protects internal electrical components and mechanical parts, prolongs the service life of equipment, and maintains stable operation. In harsh environments such as humid, dusty, or underwater conditions, motors need to adopt special sealing designs (such as rubber O-rings, corrosion-resistant stainless steel housings or bellows seals) to isolate environmental erosion and adapt to temperature and pressure changes. A good sealing protection system not only improves the environmental adaptability of motors, but also reduces maintenance costs, providing technical support for the wide applications in fields such as industrial automation, spacecraft, and underwater equipment.

There are several aspects to the design of the sealing structure, which are experiments [3,4,5,6], theoretical calculations [7,8,9], and finite element analysis [10,11].

**Figure 2:**
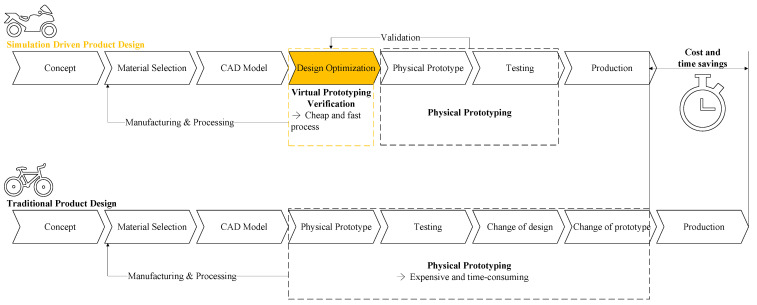
Simulation-driven product design.

Finite element analysis has emerged with the development of computer technology [12,13]. The advantages of finite element analysis are reflected in the following aspects: Firstly, its powerful ability to model complex structures allows irregular geometries to be decomposed into simple elements. By combining and analyzing the overall behavior, it can effectively handle complex boundary conditions and predict fluid mechanics that are difficult to analyze in engineering [14,15,16]. Secondly, high-precision calculations based on the equations of continuum mechanics can accurately predict parameters such as stress, strain, and displacement. It could also support multi-physics field coupling analysis (such as thermal–mechanical and fluid–structure coupling), providing comprehensive solutions for interdisciplinary problems. Thirdly, this method can replace a large number of physical experiments with virtual simulations, significantly reducing design costs and shortening the development cycle. At the same time, it enables multi-scheme optimization and comparison in the design stage, improving product reliability and performance. Figure 2 shows the comparison between traditional product design and simulation-driven product design, which can save time and costs in the design phase. In addition, a flexible meshing strategy balances efficiency while ensuring calculation accuracy, making it suitable for various problems from simple rods to three-dimensional solids. Finally, its wide range of application scenarios covers fields such as mechanics, civil engineering, aerospace, and biomedical engineering, making it a core technical support for modern engineering design and optimization [17,18].

However, as the finite element models and the physical situations to be simulated become increasingly complex, this method has gradually revealed its unique limitations. For example, in the case of material nonlinearity problems, its computation time and model convergence stability are very poor, requiring very precise adjustment of the quality of mesh division. Another example is that, for multi-contact pair problems, the mesh penetration problem has occurred frequently and it always take a very long convergence time. The current solution is to increase computing resources, such as using more CPUs for parallel computing. But this also leads to a rapid increase in simulation costs. Its timeliness and the consumption of computing resources seriously hinder the speed of product development. Simply increasing the computation time and the number of CPUs cannot solve the bottleneck problems of the finite element method.

With the explosive growth and development of artificial intelligence technology, a new solution is provided to solve this problem.

The origin of artificial intelligence can be traced back to the 1950s. The concept of “artificial intelligence” was first proposed and the discipline framework was established at the Dartmouth Conference in 1956. The early development focused on symbolism and expert systems, simulating human decision-making through rules. The proposal of the deep learning concept in 2006 and the improvement in computing power promoted the technological renaissance [19,20,21]. Landmark applications such as AlphaGo have verified the ability to solve complex tasks. Recently, artificial intelligence has penetrated into medical care (precise diagnosis and drug research and development), intelligent manufacturing (smart factories and predictive maintenance of equipment), smart cities (traffic flow optimization and AI services for government affairs), and cross-domain innovation driven by large models (such as financial risk control and educational assistance).

The development of artificial intelligence depends on three elements: data, algorithms, and computing power. Artificial intelligence is based on two major theoretical cornerstones: symbolism (logical reasoning and expert systems) and connectionism (bionic neural networks). In recent years, machine learning (especially deep learning) has become the mainstream paradigm, achieving pattern recognition and decision optimization through data-driven model training.

Machine learning is an interdisciplinary field that aims to simulate human learning behaviors in a data-driven manner and optimize the performance standards of computer programs [22]. Its theoretical roots can be traced back to the 17th century statistical foundations such as Bayes’ theorem and the least squares method, while the modern discipline framework was officially established at the Dartmouth Conference in 1956. Early development went through the exploration of symbolism and connectionism. In 1959, the self-learning checkers program designed by Samuel first verified the feasibility of machines improving the capabilities through experience, marking the transition of machine learning from theory to practice. Subsequently, during the revival period in the 1980s, expert systems and example induction learning promoted breakthroughs in automatic knowledge acquisition technology. Since the 21st century, deep learning has witnessed an explosion due to the leap in computing power and data scale. The breakthrough of AlexNet in the ImageNet competition in 2012 and the milestone event of AlphaGo demonstrated the potential of neural networks in complex pattern recognition and decision optimization. Currently, machine learning has penetrated core areas such as healthcare (e.g., imaging diagnosis and drug research and development), finance (risk prediction), transportation (autonomous driving and route planning), intelligent manufacturing (industrial robot control and predictive maintenance of equipment), and natural language processing (text generation and sentiment analysis driven by large models). It continues to expand the application boundaries through technologies such as federated learning and reinforcement learning. This technological evolution has not only reshaped the industrial ecosystem but also become the core driving force for global intelligent transformation and sustainable development, with practical effects such as reducing industrial energy consumption by an average of 15% per year.

This paper proposes hybrid models of machine learning that contain polynomial regression [23,24] and optimization XGBOOST models to rapidly and accurately predict the sealing performance of motors [25,26,27]. Then, the hybrid model is combined with the simulated annealing algorithm [28,29,30] and multi-objective particle swarm optimization algorithm for optimization. The reliability of the results is verified by the mutual verification of the results of the simulated annealing algorithm and the particle swarm optimization algorithm [31,32,33].

There is little research on the sealing structure design of motors. Most of the research focuses on the design of sealing structures.

Rubber sealing structures are sealing devices based on the elastic deformation characteristics of rubber materials [34]. The core function is to achieve fluid isolation and shock absorption protection by filling gaps or compensating for deformations [35,36]. Rubber seal origin can be traced back to the commercial application of natural rubber in the 19th century. The embryonic form of modern sealing technology was formed during the Industrial Revolution in the early 20th century. In the 1900s, engineers used annular rubber parts to solve mechanical leakage problems. Subsequently, the O-ring became a standardized product because its circular cross-section optimized the mechanical properties. Through material innovation and process iteration, rubber sealing structures achieved a technological leap in the mid-20th century. This thriving development is mainly reflected in several aspects. Breakthroughs in the research and development of synthetic rubber (such as nitrile rubber and acrylonitrile butadiene rubber) [37], multi-layer composites [38,39,40], and metal support designs significantly improved the temperature resistance and pressure-bearing capacity [41]. This promoted the application from traditional industries to high-precision fields such as aerospace [42,43] and industrial equipment [44,45,46] and even hydrogen environments [47,48,49].

Recent applications have exhibited multi-dimensional innovations. In new energy vehicles, ethylene propylene diene monomer (EPDM) sealing strips ensure the airtightness of battery packs. In the submarine field, silicone sealing rings are adopted to meet submarine pipeline maintenance dry cabin requirements [50]. In the large diameter shield machine industry, the seal is utilized to enhance the waterproof life of high-pressure and high-humidity environments [51]. According to statistics, the global rubber sealing parts market scale will surpass 100 billion US dollars by 2025. China promotes the research and development of bio–based rubber through the 14th Five-Year Plan, which verifies that this structure has become an indispensable core component in the modern industrial system.

## 2. Methodology

Polynomial regression extends linear regression by introducing high-order terms of predictor variables, enabling it to model non-linear relationships without relying on complex models. Unlike “black-box” models such as neural networks, polynomial regression provides transparent coefficients, clearly showing the contribution of each term to the prediction. Compared with support vector regression (SVR) or neural networks, it has lower requirements for computing resources and is especially suitable for datasets of medium complexity [24,52].

XGBoost outperforms SVR and random forests in terms of performance, while polynomial regression remains the preferred tool for simple non-linear modeling, considering polynomial regression and XGBoost characteristics of accuracy, efficiency, and interpretability. Therefore, polynomial regression is preferentially selected in this study, and when the prediction accuracy is poor, the XGBoost model is used for the data [53,54,55].

Import a CSV file with 119 data entries into PyCharm 2024.2.1 (Community Edition) and establish a relationship between the independent variables and the dependent variable in the finite element model of the motor sealing structure. In this study, the independent variables are preload and H (groove depth), the dependent variables are CP (contact pressure), CA (contact area), and σ (stress).

In the traditional way, the dependent variables are calculated with the FEA method through software platforms such as ANSYS workbench 2023R2. The different structure size such as H and different boundary condition such as preload are independent variables which define different FEA models and simulate through ANSYS so as to obtain the dependent variables like CP, CA, and σ. For the material non-linear problems and multi-contact problems, it usually takes a significant amount of time and occupies high CPU and RAM computing resources. Furthermore, the finite element model constructed usually has a relatively high risk of crashing during the calculation process. This risk is caused by various situations, and the most important factor is that, for material non-linearity problems, mesh distortion leads to local stress singularity, which in turn causes the calculation to crash.

In this study, by constructing the association between independent variables and dependent variables using the fitting algorithm of machine learning, two aspects of problems can be solved. On the one hand, it can address the issue of excessively long-time consumption in the finite element analysis of material non-linearity problems. On the other hand, it can solve the stability problems of the finite element simulation analysis model, such as crashes or convergence issues during the calculation process.

**Figure 3:**
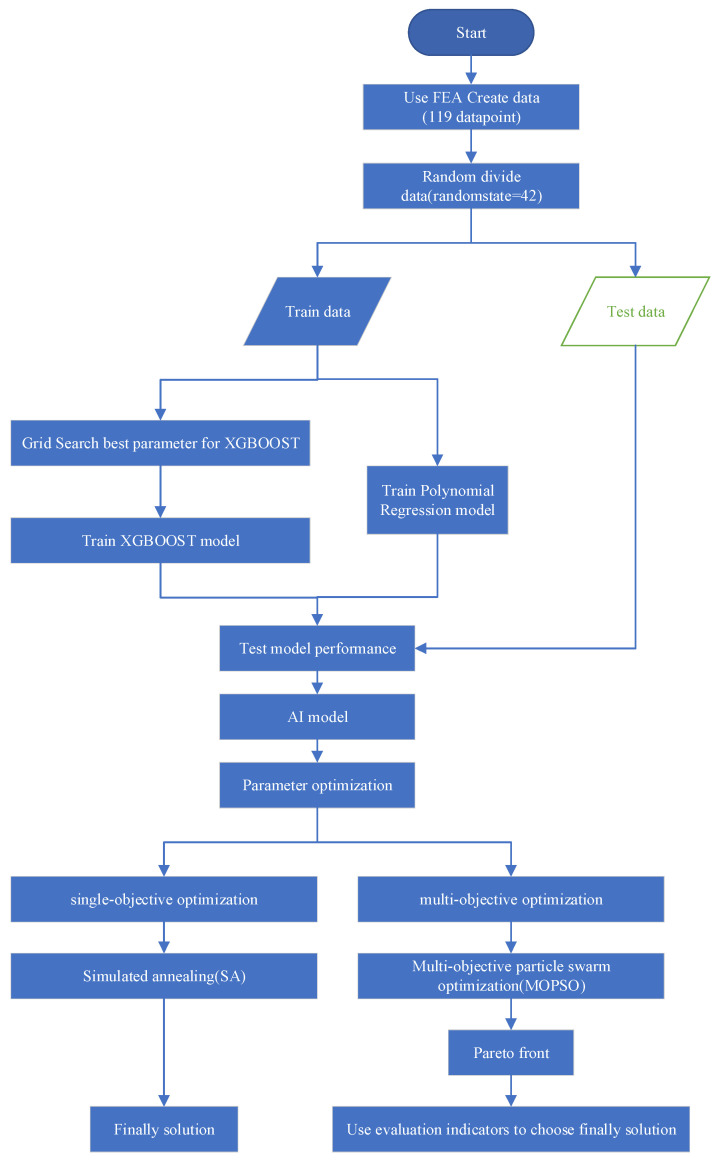
Flowchart diagram of the method.

The flowchart about the method in this study is shown in Figure 3.

### 2.1. Physical Model

**Figure 4:**
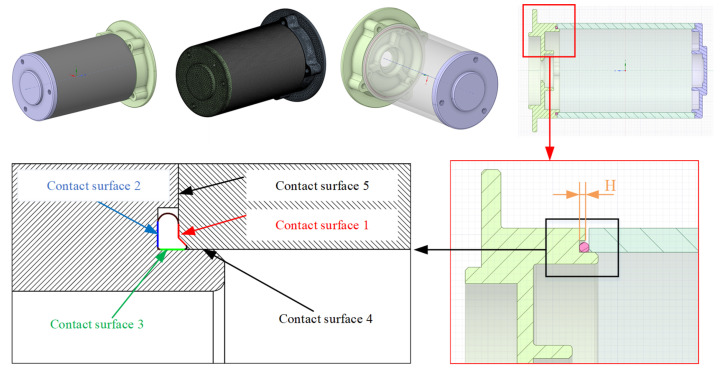
Motor model with rubber seal.

Figure 4 shows the simple motor 3D model with the O-ring, base, and shell. The section plan shows that there are three contact surfaces of the O-ring among the sealing structure, which has five contact surfaces. It also shows the FEA model, which contains the mesh element.

**Figure 5:**
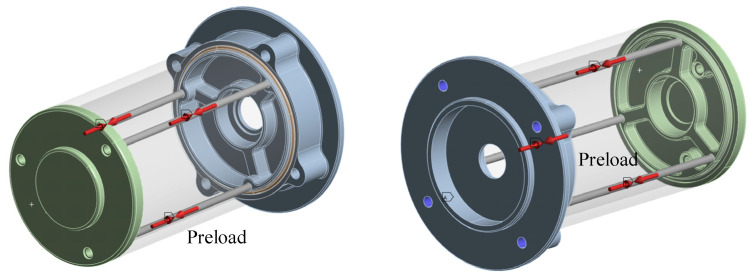
Boundary condition of FEA model.

The FEA boundary condition implies to fix the blue surface using the beam element to apply preload, which is shown in Figure 5. The FEA model is the same as in the previous study [56].

### 2.2. Polynomial Regression

Polynomial regression is a specific algorithm in machine learning. It is an extension of linear regression and is used to handle non-linear data relationships. As a non-linear relationship modeling method, polynomial regression has its historical origins tracing back to the early 19th century when Gauss and Legendre proposed the theory of least squares parameter estimation. Even earlier, the idea of nonlinear analysis stemmed from the Babylonians’ practice of using geometric methods to solve quadratic equations around 2000 BCE. The core of this algorithm lies in extending linear models into polynomial forms by introducing higher-order terms of independent variables, thereby fitting complex data patterns while maintaining the linear characteristics of parameter estimation. Its flexibility is reflected in its ability to adapt to varying data complexities through adjustments in the polynomial degree. However, high-degree models are prone to overfitting, necessitating the use of cross-validation and regularization techniques to balance the model’s generalization ability. In modern applications, polynomial regression has been extensively utilized in industrial scenarios such as semiconductor chip performance modeling, new energy battery life prediction, and automotive component reliability analysis. In terms of technical evolution, by integrating feature engineering methods such as orthogonal polynomials and spline functions, as well as machine learning frameworks like scikit-learn, the algorithm has significantly enhanced high-dimensional data processing and large-scale computational efficiency while retaining the advantages of classical statistics.

In this study, the independent variables are H (groove depth) in Figure 4 and preload. Therefore, a bivariate *n*-degree polynomial is adopted. The formula could be described as follows:

(1) $$y=\beta_{0}+\sum_{i+j\leq n}\beta_{ij}x_{1}^{i}x_{2}^{j}+\varepsilon$$

*x*_1_ and *x*_2_ are independent variables (e.g., groove depth, preload). $\beta_{0}$ is the intercept term (baseline value of *y* when all *x*-values are zero). $\beta_{ij}$ is the coefficient for interaction terms, representing the combined effect of $x_{1}^{i}$ and $x_{2}^{j}$. *n* is maximum polynomial degree (e.g., *n* = 2 for quadratic terms). ε is error term. *i* and *j* are non-negative integers representing the power exponents of two independent variables *x*_1_ and *x*_2_.

To evaluate the predictive performance of artificial intelligence models, the following metrics are used: mean absolute error (MAE), mean squared error (MSE), and mean absolute percentage error (MAPE).

The mean absolute error (MAE) is a metric used to evaluate the accuracy of predictive models, defined as the average of the absolute deviations between observed values and predicted values. Its mathematical formula is as follows:

(2) MAE=1n∑i=1nyi−yi^

where $y_{i}$ represents the true value, yi^ denotes the predicted value, and *n* is the number of samples.

A key advantage of MAE is its robustness: by utilizing absolute errors rather than squared errors, it avoids cancellation of positive and negative deviations and exhibits lower sensitivity to outliers. Compared to the mean squared error (MSE), MAE preserves the same unit as the original data, providing an intuitive measure of prediction error magnitude.

Mean squared error (MSE) is a fundamental metric in statistics and machine learning for quantifying the discrepancy between predicted values and true values.

Its mathematical formula is as follows:

(3) MSE=1n∑i=1n(yi−yi^)2

Mean absolute percentage error (MAPE) is an important indicator to measure the accuracy of a prediction model. It is used to reflect the relative error level between the predicted value and the true value. The calculation of MAPE is based on the absolute error of each sample relative to the true value. The percentage is then averaged. Its mathematical expression is as follows:

(4) MAPE=1n∑i=1nyi^−yiyi×100%

**Figure 6:**
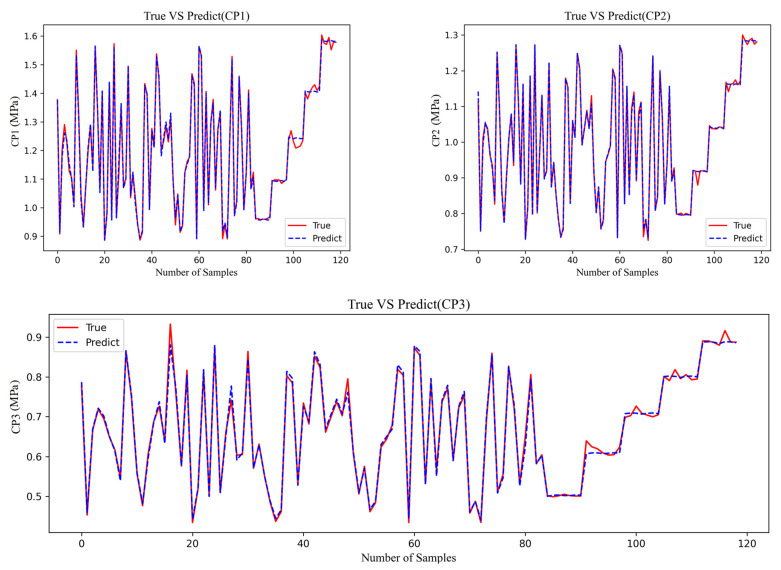
Comparison of predicted results and true values of CP.

The results of using polynomial regression simulation analysis on the database are shown in Figure 6.

By comparing the data in the figure, three evaluation methods are introduced into the data to assess the accuracy of the model. The compared results are shown in Table 1.

**Figure 7:**
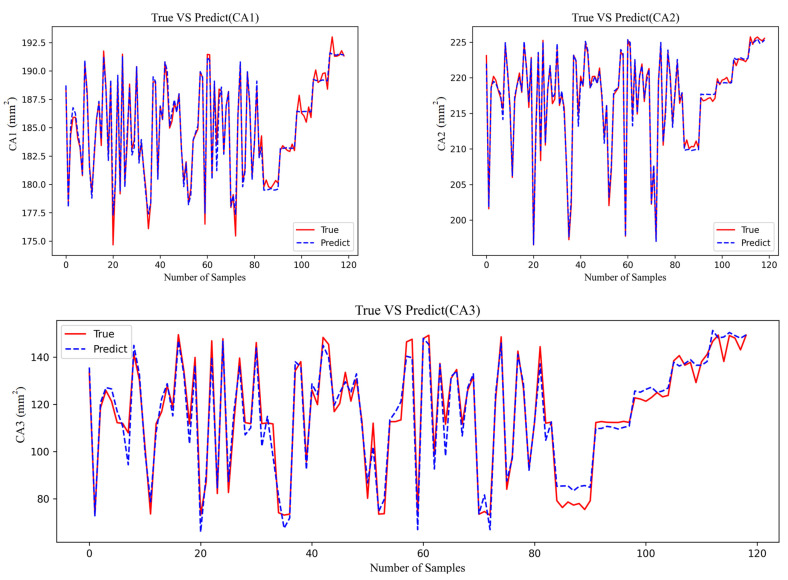
Comparison of predicted results and true values of CA.

Figure 7 shows the results of polynomial regression and the true data. For the data about CA1 and CA2, the predicted results are highly reliable while CA3 is not satisfactory. The MES of CA3 reaches 39.82, which is the highest value among all the CA results in Table 2.

**Figure 8:**
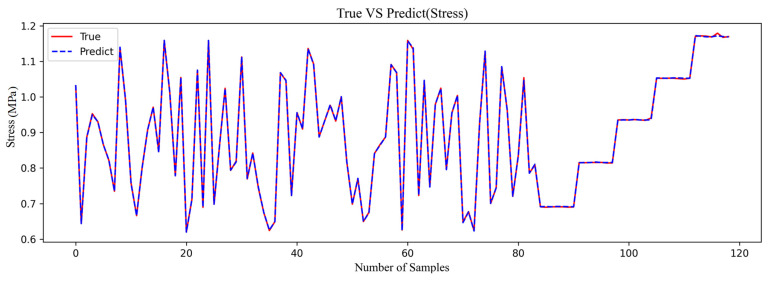
Comparison of predicted results and true values of stress.

Figure 8 shows the results of polynomial regression and the true data. For the data about O-ring stress, the predicted results are highly reliable. The MAPE is 0.1257% in Table 3.

The above analysis shows that, except for the CA3 indicator, the fitting performance of other metrics is satisfactory. In order to improve the fitting accuracy and make subsequent parameter optimization more reliable, the advanced model XGBOOST in machine learning will be used.

### 2.3. XGBOOST

XGBoost (eXtreme Gradient Boosting) is an ensemble learning algorithm based on gradient boosting decision trees. It iteratively optimizes model performance by combining the prediction results of weak learners. Its core improvement lies in the introduction of second-order Taylor expansion and regularization terms to balance model complexity and generalization ability. This algorithm supports parallel computing and distributed processing, and adopts greedy splitting strategy and cache-aware optimization technology, which significantly improves the training efficiency on large-scale data. At the same time, it realizes feature importance analysis through interpretable frameworks such as SHAP (Shapley Additive Explanations). The flexibility and scalability of XGBoost enable it to support custom loss functions and evaluation metrics, providing a general solution for machine learning tasks in complex scenarios.

After fitting with polynomials, the fitting effect on the physical quantity CA3 is not satisfactory. In this section, another machine learning algorithm is introduced. XGBOOST is used to improve the accuracy. The trained XGBOOST is compared with the polynomial model in the same test set. Calculate the error metrics for the XGBOOST model that are MSE, MAE, and MAPE. The calculation results of the error metrics for the XGBOOST model are in Table 4.

Compared with Table 2, for the error metrics of quantity CA3, the XGBOOST model has a better performance than the polynomial regression model. For the model accuracy purpose, although the XGBOOST model is better, it still needs to improve the accuracy. To improve the fitting effect of XGBOOST, use Grid Search to optimize its parameters. Grid Search works by providing several sets of model parameters, aiming to find the optimal parameter combination so that the model performs better on the test set. Grid Search in XGBoost is a systematic hyper-parameter optimization method. Its core is to traverse the pre-defined hyper-parameter combinations through exhaustive search, and combine cross-validation to evaluate model performance, so as to select the optimal parameter configuration. This method requires pre-defining the parameter search space (such as learning rate, tree depth, etc.), generating all possible parameter combinations, and calculating the average performance metrics (such as log loss, accuracy) through cross-validation to determine the best parameters. The advantage of grid search lies in its global optimality, ensuring that the best parameters are found within the given search space. Perform grid search and cross-validation using Grid Search on the training set to find the optimal parameters and the corresponding model, and calculate the error metrics on the test set. The optimization process culminated in the successful identification of the relevant parameters following a 43 min exploration. The error metrics of the optimized XGBOOST model are listed in Table 5. Compared to the data in Table 4, for the error metric results of the optimized XGBOOST model, there has been a certain improvement for each physical quantity, indicating that the optimized model is effective. According to Occam’s Razor, which is a philosophical and scientific principle advocating for simplicity, among competing hypotheses explaining the same phenomenon, the one with the fewest assumptions should be prioritized. For cases wherein the accuracy of polynomial fitting is not significantly different from that of OP-XGBOOST, polynomial regression is still used. For cases with significant improvement, OP-XGBOOST is employed.

**Figure 9:**
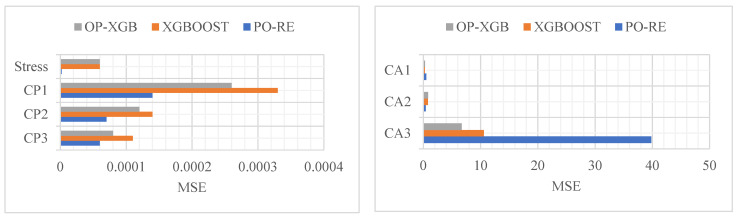
Comparison of prediction performance based on MSE.

**Figure 10:**
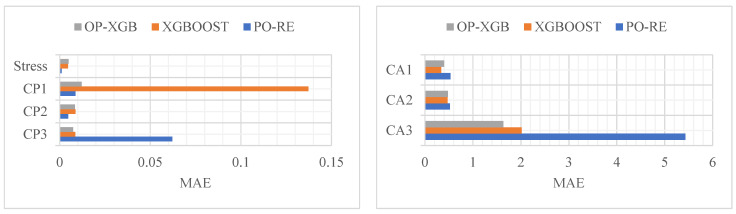
Comparison of prediction performance based on MAE.

**Figure 11:**
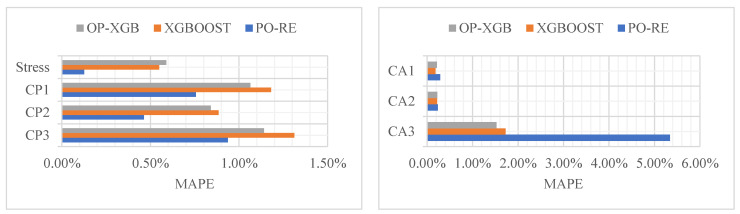
Comparison of prediction performance based on MAPE.

Analyze the data from Table 1, Table 2, Table 3, Table 4 and Table 5 to obtain the bar chart from Figure 9, Figure 10 and Figure 11. The comparison of the prediction results of CP and stress reveals that PO-RE has a better performance than the other comparative models of MSE. While CA has a better performance of OP-XGB, the PO-RE model’s MSE reaches 39.82. The comparison of MAE and MAPE of CP and stress indicate that the PO-RE model is better than the OP-XGB model besides the MAE of CP3. For the CP3, the MAE of the PO-RE model is 0.06223 while that of OP-XGB is 0.00744. The comparison of the prediction results of CA reveals that OP-XGB has a better performance than the PO-RE models.

According to the above analysis, the final model is established in Table 6. That is, for CP and stress, polynomial regression is better, while for CA, OP-XGBOOST is preferred.

**Figure 12:**
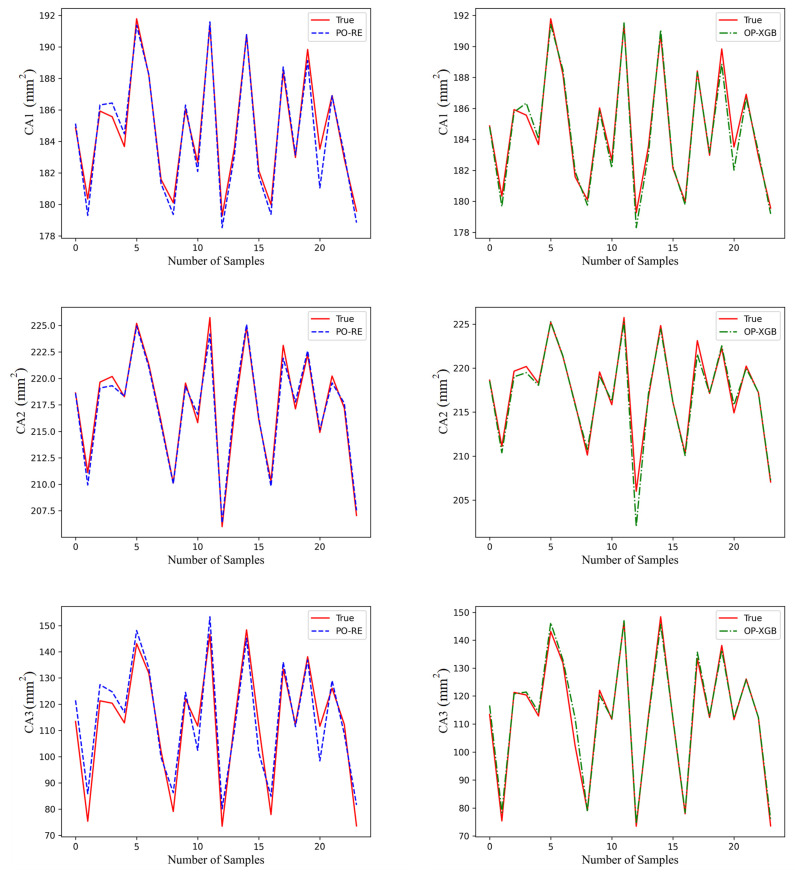
Comparison of prediction performance of CA.

**Figure 13:**
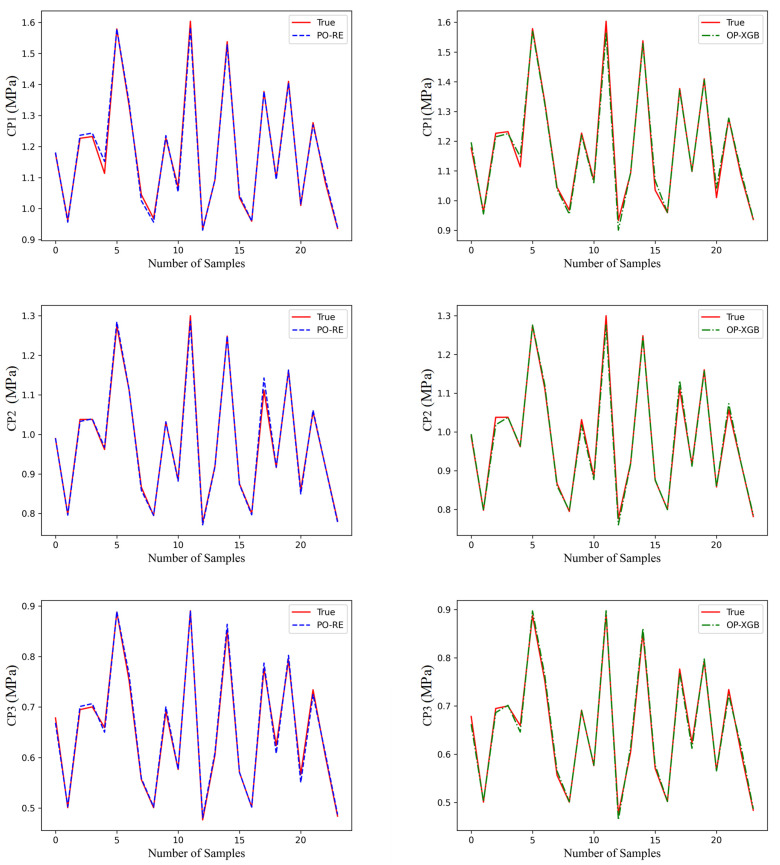
Comparison of prediction performance of CP.

**Figure 14:**
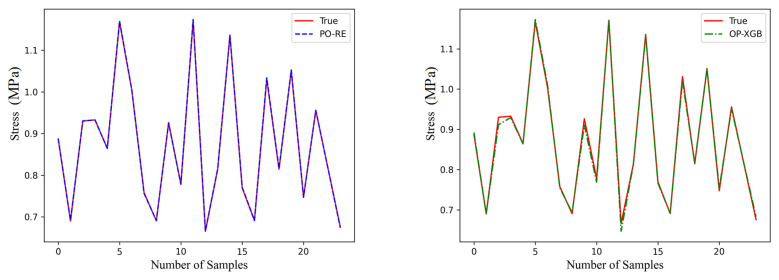
Comparison of prediction performance of stress.

Validate the above models on the validation set. Save the above-generated models as DAT files, and then applied the models to fit the data on the validation set. The performance of the different models is shown in Figure 12, Figure 13 and Figure 14.

The red line is original data from the test set. The blue line, named PO-RE, is polynomial regression. The green line, named OP-XGB, is Optimized-XGBOOST.

From the comparison of CP in Figure 13, it indicates that the accuracy difference between the models using OP-XGBOOST and polynomial regression is small. The performance of the polynomial regression model is slightly better than that of OP-XGBOOST, and it is generally closer to the original data. Therefore, the polynomial regression model is used for the CP physical quantity.

From the fitting effect of CA in Figure 12, it indicates that the error of fitting CA using polynomial fitting is relatively large, which is obvious in CA2 and CA3. However, the fitting error of using OP-XGBOOST is smaller, and the fitting curve is closer to the original data curve. Therefore, for the CA physical quantity, OP-XGBOOST is used for fitting.

By comparing the performance of two different machine learning models in fitting stress in Figure 14, it can be concluded that both polynomial fitting and XGBOOST perform well. However, the polynomial fitting result is closer to the original data. Therefore, for this physical quantity, the polynomial regression model is adopted.

**Figure 15:**
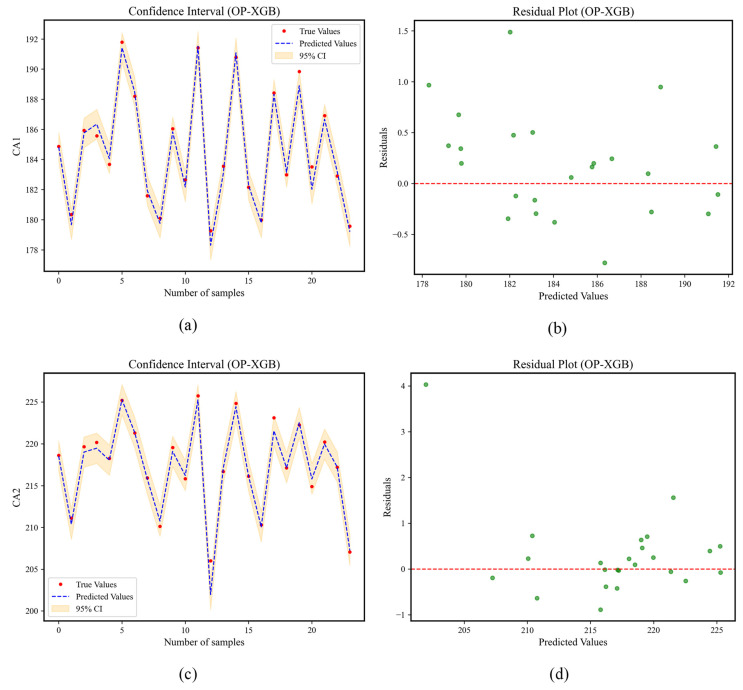

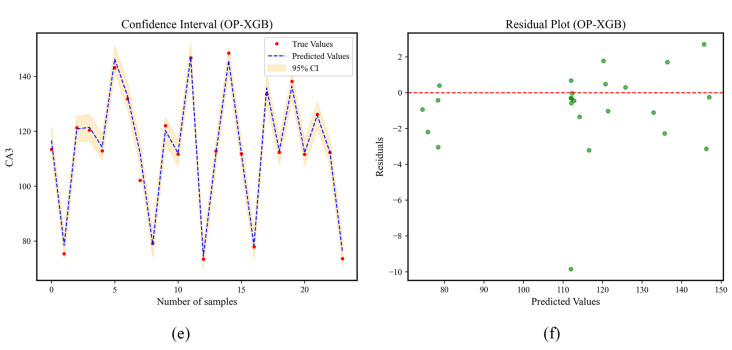
Confidence intervals and residual plots of OP-XGB. (**a**) Confidence interval of CA1, (**b**) residual plot of CA1, (**c**) confidence interval of CA2, (**d**) residual plot of CA2, (**e**) confidence interval of CA3, (**f**) residual plot of CA3.

The OP-XGBOOST confidence intervals and residual plots are shown in Figure 15. In Figure 15a, the red dots represent the actual values, the blue dashed line represents the sequence of predicted values of the model for the test set, and the orange confidence band is the 95% prediction interval calculated based on the standard deviation of the residuals. It can be seen from the figure that the red dots are evenly distributed around the blue line, and most (≥95%) of the red dots are located within the orange band. Moreover, the confidence band is narrow and uniform. In Figure 15b, the data points are randomly distributed around the red line of y = 0 without any regular pattern.

**Figure 16:**
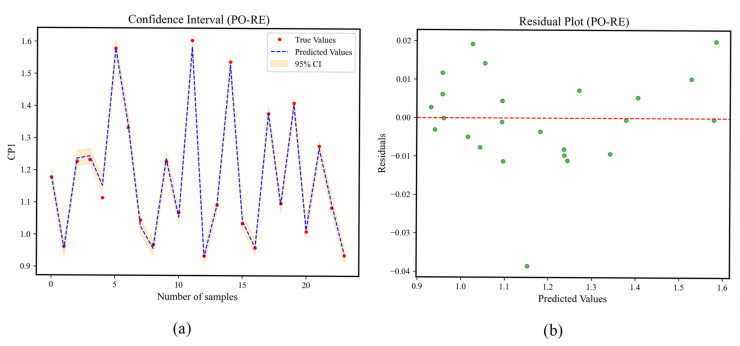

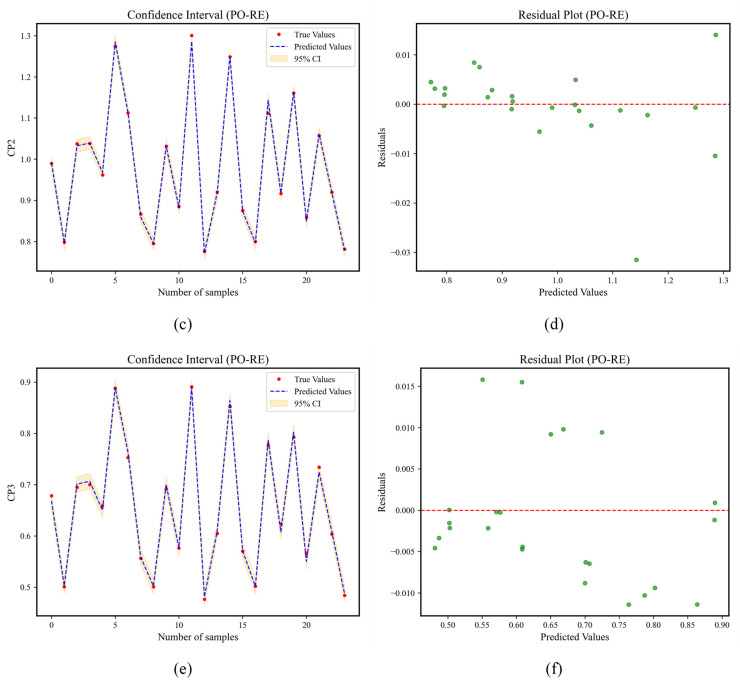
Confidence intervals and residual plots of PO-RE. (**a**) Confidence interval of CP1, (**b**) residual plot of CP1, (**c**) confidence interval of CP2, (**d**) residual plot of CP2, (**e**) confidence interval of CP3, (**f**) residual plot of CP3.

The PO-RE confidence intervals and residual plots are shown in Figure 16. In Figure 16a, the red dots represent the actual values, the blue dashed line represents the sequence of predicted values of the model for the test set, and the orange confidence band is the 95% prediction interval calculated based on the standard deviation of the residuals. It can be seen from the figure that the red dots are evenly distributed around the blue line, and most (≥95%) of the red dots are located within the orange band. Moreover, the confidence band is narrow and uniform. In Figure 16b, the data points are randomly distributed around the red line of y = 0 without any regular pattern.

**Figure 17:**
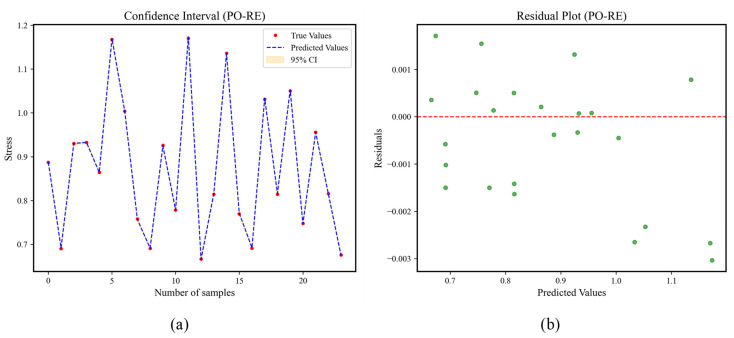
Confidence intervals and residual plots of PO-RE. (**a**) Confidence interval of stress, (**b**) residual plot of stress.

The PO-RE confidence intervals and residual plots are shown in Figure 17. In Figure 17a, the red dots represent the actual values, the blue dashed line represents the sequence of predicted values of the model for the test set, and the orange confidence band is the 95% prediction interval calculated based on the standard deviation of the residuals. It can be seen from the figure that the red dots are evenly distributed around the blue line, and most (≥95%) of the red dots are located within the orange band. Moreover, the confidence band is narrow and uniform. In Figure 17b, the data points are randomly distributed around the red line of y = 0 without any regular pattern.

### 2.4. Simulated Annealing Algorithm

The simulated annealing algorithm is a global optimization algorithm based on the principle of solid annealing. By simulating the process of a substance being heated and then slowly cooled, it randomly searches for the global optimal solution of the objective function in the solution space. Its core idea is to use the Metropolis criterion to accept inferior solutions with a certain probability at each temperature, so as to jump out of the local optimal trap and finally converge to an approximately optimal solution at low temperatures. The algorithm includes five key steps: initialization, neighborhood search, objective function evaluation, acceptance criterion judgment, and temperature attenuation. By controlling parameters (such as initial temperature and cooling rate), it balances exploration and exploitation.

This study references the previous research, empirical data, and GB/T 3452.3-2005 [56,57]. The upper and lower limits of groove depth and preload are set to [0.6, 0.9] and [70, 80], respectively. The initial solution is set to [0.75, 75], where the groove depth is 0.75 mm and the preload is 75 N. Since the principle of the simulated annealing algorithm is to find the minimum value, the object is set to

(5) $$Object=min\;[-\left(cp3+cp2+cp1+ca3+ca2+ca1-stress\right)]$$

Three objective functions are set, namely Object1, Object2, and Object3; that is,

(6) $$Object1=\sum_{i=1}^{3}{Cp}_{i}$$

(7) $$Object2=\sum_{i=1}^{3}{Ca}_{i}$$

(8) Object3=Stress

(9) Function=Max (Object1)+Max (Object2)+Min (Object3)

For the SA algorithm, it uses the model generated by the machine learning algorithm to optimize data. The list of the results of data optimization is found in Table 7. The optimal solutions are all close to point (0.8, 80), with the groove depth near 0.8 mm and the preload from 79 N to 80 N.

### 2.5. Multi-Objective Particle Swarm Optimization

Multi-objective particle swarm optimization (MOPSO) is an extension of the classic particle swarm optimization (PSO) algorithm, designed to address multi-objective optimization problems where conflicting objectives need to be simultaneously optimized. Originating from the social behavior of bird flocks, MOPSO simulates the collective movement of particles to explore the solution space, guided by both individual and global best position.

The particle swarm optimization (PSO) algorithm simulates the process of birds searching for food in the forest. During the food-searching process, the velocity of a bird is determined by three aspects: its own inertia, self-cognition, and group-cognition. These three aspects determine the magnitude and direction of the velocity.

(10) $$V_{i+1}=wV_{i}+r_{1}c_{1}\left(Pbest-X_{i}\right)+r_{2}c_{2}(Gbest-X_{i})$$

Among them, w is Inertia weight, *r*_1_ and *r*_2_ are random numbers between [0, 1], $c_{1}$ and $c_{2}$ are the cognitive and social acceleration coefficients, *Pbest* is the particle’s own historical optimal solution, and *Gbest* is the best position that has been found by the whole swarm [58].

(11) $$X_{i+1}=X_{i}+V_{i}$$

Particle swarm optimization is commonly used to deal with single-objective functions. The magnitude of the function value is represented by fitness. Different positions of the small bird correspond to different fitness values; that is, different function values. Since it is a single-objective function, it is very easy to judge the quality of the particle’s position and determine whether to perform this update according to the magnitude of the updated function value.

In multi–objective particle swarm optimization (MOPSO), a simple approach is that, each time the particle position is updated, if the updated solution dominates the original solution, the replacement is carried out; otherwise, the updated solution is added to the non-dominated solution set, and one is randomly selected as Pbest and gbest.

**Figure 18:**
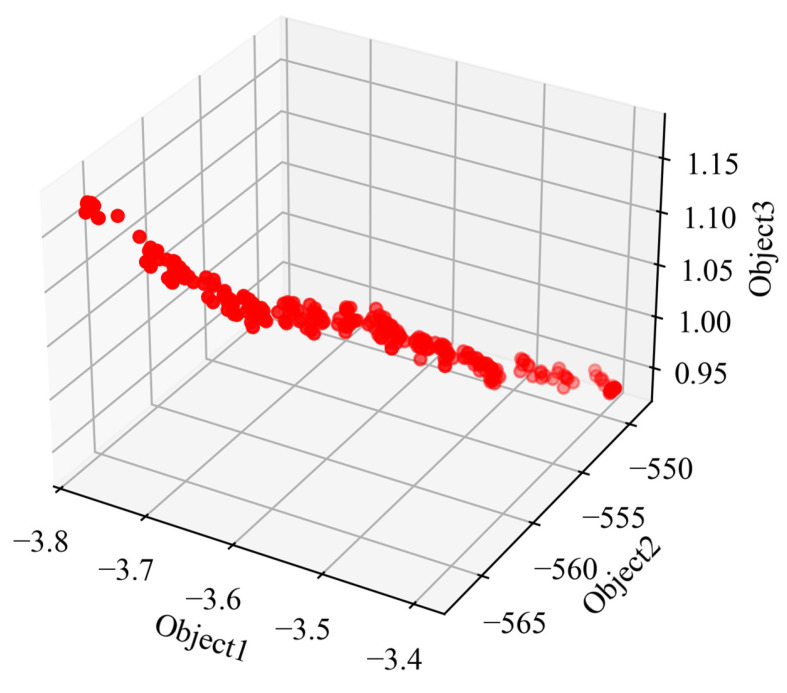
Pareto front.

In this study, the objective function is Object1, Object2, and Object3. Define the initial population set to 300, and the final obtained Pareto front is shown in Figure 18.

The points on the Pareto front are all non-dominated relationships; that is, there is no absolute superiority or inferiority relationship, which can provide multiple choices rather than being completely based on the weighted sum value. It can also be seen from the figure that, when Object1 and Object2 are larger, the stress is also large; if the stress were to be smaller, Object1 and Object2 would also become smaller. Pareto front database is listed in Table 8.

**Figure 19:**
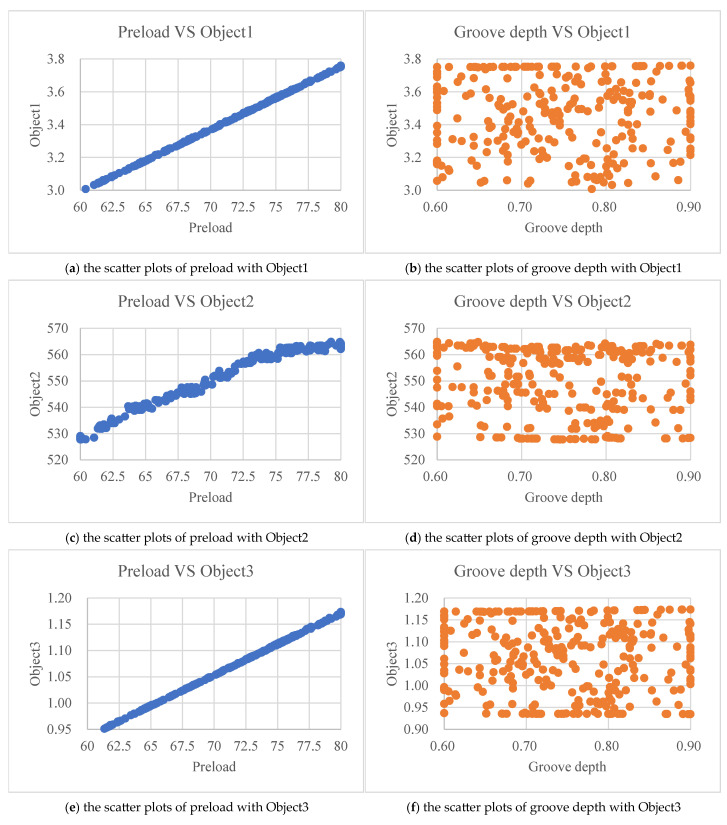
Distribution characteristics of variables.

Construct scatter plots of preload with Object1, Object2, and Object3 for further analysis in Figure 19. A good linear relationship is shown between preload and Object1. At the same time, a good linear relationship is also shown between preload and Object3. A synchronous increasing relationship is shown between preload and Object2. On the contrary, groove depth with Object1, Object2, and Object3 display a random distribution, with data points disorderly scattered and no obvious correlation between variables.

From the relationship graph among preload, groove depth, and Object, it can be concluded that the magnitude of Object is determined by the magnitude of preload, and the influence of groove depth on Object is randomly distributed without any regular pattern.

According to the GB/T 3452.3-2005 and SA result, screen out the points from the Pareto front wherein the Groove depth is between [0.8, 0.85]. There is a total of 26 design schemes after screen out. Conduct a comprehensive evaluation of these 26 design schemes again. The top five results after evaluation using principal component analysis (PCA) are shown in Table 9. The main steps of principal component analysis (PCA) include data standardization, calculation of the covariance matrix, eigenvalue decomposition, selection of principal components, allocate weights based on the variance contribution rate of principal components, project the data into the principal component space, calculate the comprehensive scores, and then obtain rank [59,60,61].

## 3. Results and Discussion

The purpose of this study is to intelligently find a set of optimal sealing structure design parameters through artificial intelligence methods, to design the optimal solution in the virtual design stage.

### 3.1. Optimize the Best Design Solution

This research combines machine learning algorithms and optimization algorithms to optimize the motor’s waterproof sealing design scheme. The aim is to find the optimal structural dimensions during the virtual prototype design stage.

For the SA method, the optimal parameter is groove depth H = 0.8 mm with preload 79 N in Table 7. For the MOPSO method, the optimal parameter is H = 0.85 mm with preload 80 N in Table 9. The range of H is from 0.8 mm to 0.85 mm among the top five rank with the preload 80 N.

Therefore, for the optimal design structure in this study, the optimal H is from 0.8 to 0.85 mm with the preload from 79 N to 80 N.

### 3.2. AI Model Verification

In order to verify the accuracy of the machine learning model again, finite element analysis is carried out on special data outside the training data samples, and the trained machine learning model is used for prediction. The preload is 90 N while the groove depth H of 0.9 mm is defined as the special data. The results are shown in Table 10.

**Figure 20:**
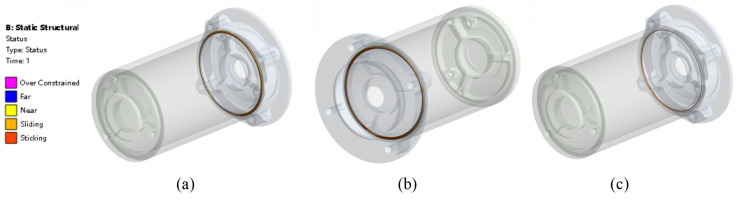
O-ring contact status. (**a**) CA1 contact status, (**b**) CA2 contact status, (**c**) CA3 contact status.

**Figure 21:**
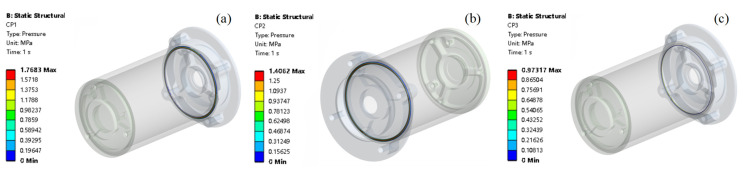
O-ring contact pressure. (**a**) CP1 contact pressure, (**b**) CP2 contact pressure, (**c**) CP3 contact pressure.

The contour plot from Figure 20 shows the contact status of different contact surfaces. Figure 21 shows the contact pressure of different contact surfaces, Figure 21a is the contact pressure of contact surface1 for which the max contact pressure is 1.768 MPa. Figure 21b is the contact pressure of contact surface2 for which the max contact pressure is 1.406 MPa. Figure 21c is the contact pressure of contact surface3 for which the max contact pressure is 0.973 MPa.

**Figure 22:**
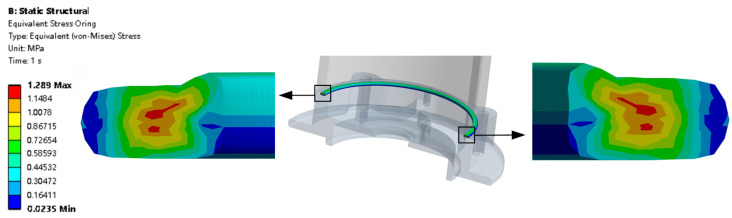
O-ring stress.

Figure 22 shows the O-ring stress distribution. The maximum stress is located at the middle part on the inside of the O-ring. The stress distribution shows a trend where the stress is relatively large in the middle and smaller on the outside.

**Figure 23:**
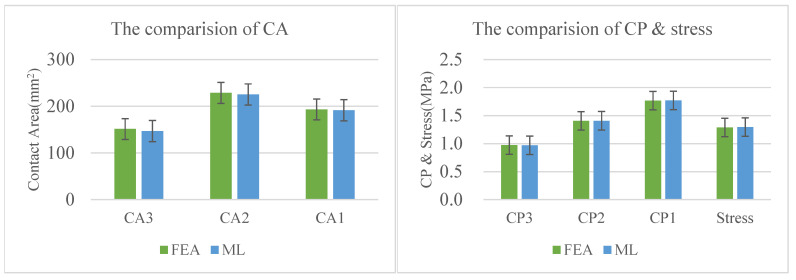
The comparison of ML and FEA methods.

For the data without training, the prediction results of machine learning demonstrated high accuracy, with a deviation range of 0.127–2.881%. For the data CP3, CA3, CA2, CA1, the prediction results of machine learning are smaller than the calculation results of FEA. In addition, for the other data, the situation is the opposite. Overall, the prediction results of machine learning fluctuate on both sides of the calculation results of FEA, with a very small deviation in Figure 23.

## 4. Conclusions

This research combines machine learning algorithms and optimization algorithms to optimize the motor’s waterproof sealing design scheme. The aim is to find the optimal structural dimensions during the virtual prototype design stage. Conclusions are summarized as follows:

(1) The XGBOOST does not have better fitting effect than polynomial regression on all data. Before using the model, it is necessary to compare different models and select an appropriate model to increase the fitting accuracy.
(2) Machine learning models not only have good prediction effects within the range of training data, but also exhibit high prediction accuracy for untrained data, such as in the case of a preload of 90 N. This verifies the applicability scope of ML models. Compared with the traditional FEA method, which incurs a high time cost of 9 h to obtain calculation results, the ML model takes less than 1 s, demonstrating extremely high efficiency. However, traditional FEM models have to be employed to obtain the training data required for machine learning.
(3) This research deeply reveals the correlation mechanism and influence laws between input parameters and the sealing performance of motor waterproof structures, and provides an important theoretical basis for optimizing sealing structure design and improving sealing performance.
(4) The analysis results through the simulated annealing algorithm show that, in the optimal design scheme, the groove depth is 0.8 mm and the preload is 79 N. In the solution set of the Pareto front obtained through the multi-objective particle swarm optimization algorithm, PCA analysis is carried out to find the optimal solution to be 0.85 mm, and the preload is 80 N. There are some differences between the two methods, but the optimal design can be obtained through cross-verification of the two algorithms. The optimal design is that the groove depth is 0.8–0.85 mm, and the preload is 79–80 N.
(5) This paper systematically evaluates the performance of machine learning models (including single algorithms and hybrid models) in predicting motor sealing performance. Relevant data and results can provide scientific guidance for designers to choose optimal design solutions according to specific needs. Research has shown that machine learning models can significantly improve the speed and accuracy of sealing performance prediction. The proposed methodology could be generalized to other mechanical systems such as sealing in aerospace and marine applications.

## Figures and Tables

**Table 1:** The error metrics of polynomial regression results.

	CP3	CP2	CP1
MSE	0.00006	0.00007	0.00014
MAE	0.06223	0.00473	0.00877
MAPE	0.9372%	0.4631%	0.7561%

**Table 2:** The error metrics of polynomial regression results.

	CA3	CA2	CA1
MSE	39.81617	0.42164	0.52227
MAE	5.43012	0.51951	0.52921
MAPE	5.3416%	0.2383%	0.289%

**Table 3:** The error metrics of polynomial regression results.

Method	MSE	MAE	MAPE
σ result	0.000002	0.00111	0.1257%

**Table 4:** The error metrics of the XGBOOST model.

	CP3	CP2	CP1	CA3	CA2	CA1	Stress
MSE	0.00011	0.00014	0.00033	10.5760	0.82313	0.26770	0.00006
MAE	0.00861	0.00872	0.13726	2.01653	0.46950	0.33981	0.00464
MAPE	1.3119%	0.8845%	1.1812%	1.7257%	0.2193%	0.1850%	0.5486%

**Table 5:** The error metrics of OP-XGBOOST.

	CP3	CP2	CP1	CA3	CA2	CA1	Stress
MSE	0.00008	0.00012	0.00026	6.69089	0.84413	0.31780	0.00006
MAE	0.00744	0.00838	0.01218	1.63360	0.47834	0.39895	0.00493
MAPE	1.1407%	0.8405%	1.0644%	1.5270%	0.2239%	0.2167%	0.5891%

**Table 6:** Model for quantity.

Variable	Model
CP3	Polynomial Regression (degree = 2)
CP2	Polynomial Regression (degree = 1)
CP1	Polynomial Regression (degree = 2)
CA3	Optimized-XGBOOST
CA2	Optimized-XGBOOST
CA1	Optimized-XGBOOST
Stress	Polynomial Regression (degree = 3)

**Table 7:** SA algorithm optimized results.

	Groove Depth (mm)	Preload (N)
1	0.80031234	79.07348483
2	0.80045546	79.85661158
3	0.79310159	79.59513457
4	0.80133705	79.57056586
5	0.80097211	79.82807542
Average	0.79923571	79.58477445

**Table 8:** Part of Pareto front database.

	H	Preload	CP3	CP2	CP1	CA3	CA2	CA1	Stress	Obj1	Obj2	Obj3
1	0.698	60.206	0.710	1.042	1.246	122.324	219.593	186.128	0.938	2.998	528.044	0.938
2	0.860	79.110	0.882	1.275	1.566	147.384	225.038	191.673	1.162	3.723	564.095	1.162
3	0.684	64.920	0.755	1.100	1.321	131.252	221.069	188.106	0.993	3.175	540.427	0.993
…	…	…	…	…	…	…	…	…	…	…	…	…
25	0.707	60.000	0.708	1.040	1.242	122.324	219.593	186.128	0.935	2.991	528.044	0.935
26	0.600	79.248	0.879	1.274	1.569	148.194	225.109	191.533	1.160	3.722	564.836	1.160
27	0.738	75.154	0.848	1.226	1.493	145.224	223.567	189.576	1.113	3.566	558.367	1.113
28	0.782	64.287	0.750	1.093	1.311	130.975	220.427	187.661	0.986	3.154	539.062	0.986
29	0.691	69.656	0.798	1.158	1.399	137.535	222.359	188.890	1.048	3.355	548.783	1.048
…	…	…	…	…	…	…	…	…	…	…	…	…
296	0.859	62.388	0.732	1.070	1.283	126.765	220.540	186.886	0.963	3.085	534.192	0.963
297	0.624	71.868	0.816	1.184	1.438	143.065	223.136	189.263	1.074	3.439	555.464	1.074
298	0.600	65.072	0.754	1.101	1.326	131.978	221.106	188.253	0.996	3.181	541.337	0.996
299	0.600	75.189	0.844	1.225	1.496	147.357	223.603	189.652	1.113	3.566	560.612	1.113
300	0.656	61.773	0.724	1.061	1.271	125.938	220.009	186.669	0.956	3.057	532.616	0.956

**Table 9:** PCA rankings of different design parameters.

Groove Depth	Preload	Score	Rank
0.847651	80	0.954222	1
0.839494	80	0.951231	2
0.807247	80	0.949080	3
0.804788	80	0.948161	4
0.801953	80	0.947103	5

**Table 10:** Comparison of ML and FEA methods.

Method	CP3	CP2	CP1	CA3	CA2	CA1	Stress
FEA	0.973	1.406	1.768	151.342	228.689	193.234	1.289
ML	0.971	1.408	1.771	146.982	225.252	191.516	1.297
Deviation	−0.002	0.002	0.001	−0.029	−0.015	−0.009	0.006
Deviation (%)	−0.245	0.152	0.127	−2.881	−1.503	−0.889	0.605

## Data Availability

The data that support the findings of this study are available from the corresponding authors upon reasonable request.

## Nomenclature

Symbol	Unit	Implication
CP	[MPa]	Contact pressure
CP1	[MPa]	Contact pressure of Contact surface 1
CP2	[MPa]	Contact pressure of Contact surface 2
CP3	[MPa]	Contact pressure of Contact surface 3
CA	[mm^2^]	Contact area
CA1	[mm^2^]	Contact area of Contact surface 1
CA2	[mm^2^]	Contact area of Contact surface 2
CA3	[mm^2^]	Contact area of Contact surface 3
σ	[MPa]	Von mises stress
H	[mm]	Groove depth
x, y, z	[m]	Cartesian coordinates
ML	[-]	Machine learning
XGBOOST	[-]	eXtreme Gradient Boosting
NBR	[-]	nitrile-butadiene rubber
eVTOL	[-]	Electric vertical takeoff and landing
FEM	[-]	Finite Element Method
PCA	[-]	Principal Component Analysis
